# High level of anti-drug antibodies is associated with shorter survival in advanced solid cancer patients treated with Immune checkpoint inhibitors

**DOI:** 10.1093/immadv/ltaf019

**Published:** 2025-06-07

**Authors:** Rui Zhao, Weihao Wang, Jingliang Wang, Yahui Wang, Liying Pan, Pancen Ran, Fang Luan, Guobin Fu

**Affiliations:** Department of Oncology, Shandong Provincial Hospital Affiliated to Shandong First Medical University, Jinan, Shandong 250021, China; Department of Clinical Laboratory, Shandong Provincial Hospital Affiliated to Shandong First Medical University, China; Department of Oncology, Shandong Provincial Hospital Affiliated to Shandong First Medical University, Jinan, Shandong 250021, China; Department of Clinical Laboratory, Shandong Provincial Hospital Affiliated to Shandong First Medical University, China; The Clinical Medical College, Shandong First Medical University (Shandong Academy of Medical Sciences), Jinan, Shandong 250117, China; The Clinical Medical College, Shandong First Medical University (Shandong Academy of Medical Sciences), Jinan, Shandong 250117, China; The Clinical Medical College, Shandong First Medical University (Shandong Academy of Medical Sciences), Jinan, Shandong 250117, China; The Clinical Medical College, Shandong First Medical University (Shandong Academy of Medical Sciences), Jinan, Shandong 250117, China; The Clinical Medical College, Shandong University of Traditional Chinese Medicine, Jinan, Shandong 250013, China; Department of Oncology, Shandong Provincial Hospital Affiliated to Shandong First Medical University, Jinan, Shandong 250021, China; Department of Clinical Laboratory, Shandong Provincial Hospital Affiliated to Shandong First Medical University, China; The Clinical Medical College, Shandong First Medical University (Shandong Academy of Medical Sciences), Jinan, Shandong 250117, China; The Clinical Medical College, Shandong University, Jinan, Shandong 250012, China; Department of Oncology, The Third Affiliated Hospital of Shandong First Medical University, Jinan, Shandong, 250031, China

**Keywords:** anti-drug antibodies, camrelizumab, immunogenicity, pd-1, advanced solid cancer

## Abstract

**Background:**

Camrelizumab has become the first-line treatment for most patients with advanced tumors. Among advanced tumor patients undergoing camrelizumab, the majority develop immunogenicity, resulting in the production of anti-drug antibodies (ADA). The impact of ADA on the efficacy and safety of camrelizumab treatment is currently unknown.

**Method:**

Hematologic samples from 31 tumor patients treated with camrelizumab were collected to serve as an experimental cohort for ADA levels detection. Concurrently, a separate validation cohort consisting of 16 patients was established. Follow-up data on patients’ OS and PFS were collected and analyzed.

**Results:**

High ADA levels (≥1200 ng/ml) after the three cycles camrelizumab treatment were linked to poorer patient outcomes, as shown by significant differences between PD and PR (*P* = 0016) and PR and SD (*P* = .0439). This trend was also present in the validation cohort (PD vs PR, *P* = .0413). More importantly, high ADA levels after the three cycles camrelizumab treatment were associated with a significant reduction in OS (*P* = .0128) and PFS (*P* = .0004), with the validation cohort reporting comparable findings (OS: *P* = .0009; PFS: *P* = .0007). Additionally, camrelizumab concentration was negatively correlated with ADA levels (experimental cohort: *R*^2^ = 0.3876; validation cohort: *R*^2^ = 0.3702). Patients had higher ADA levels after the early phase of camrelizumab treatment.

**Conclusion:**

High ADA levels were associated with shorter OS and PFS in patients after three cycles of camrelizumab therapy. Furthermore, patients had higher ADA levels after the early phase of treatment, specifically in the first three cycles with camrelizumab. It found that the higher the ADA concentration, the lower the serum camrelizumab concentration.

## Graphical Abstract

**Graphical Abstract:**
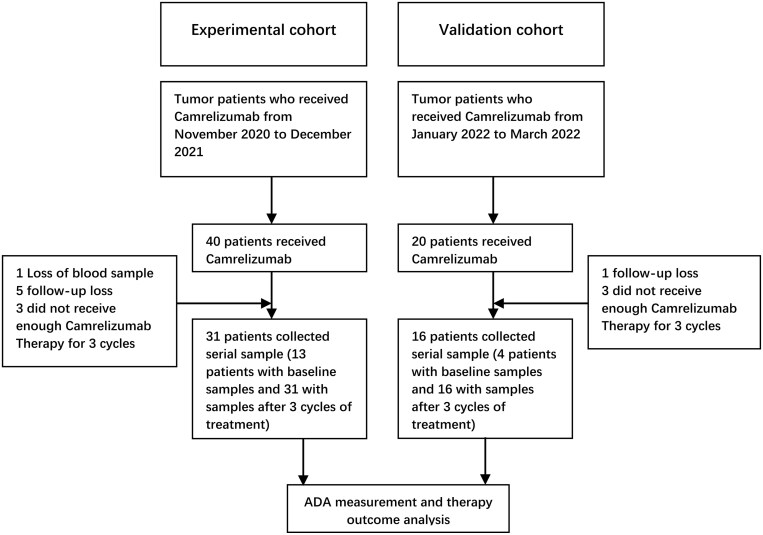

## Introduction

Immune checkpoint inhibitors (ICIs), particularly monoclonal antibodies (mAbs) that block the interaction between programmed death 1(PD-1)/programmed death ligand-1 (PD-L1), have become standard treatments for various tumors [1–3]. However, monoclonal antibody therapies can elicit immunogenicity, often triggering the production of ADA (63%) [4]. ADA is caused by the immune response to the drug. Once it enters the circulation, it may combine with the immunogenic components of the drug, which may change its pharmacokinetics and pharmacodynamics as well as its efficacy and safety [4]. The end result limits the therapeutic effect of patients receiving ICIs [5]. The US Food and Drug Administration (FDA) have issued guidelines that advocate for the assessment of immunogenicity in therapeutic proteins during the early and subsequent stages of drug development [6].

Camrelizumab, a humanized monoclonal antibody against PD-1, was approved for sale by the US FDA in 2016. In some studies, it has been reported that camrelizumab has antitumor activity against various tumor types and has acceptable safety [7–9]. Currently, camrelizumab in combination with platinum-based therapy is recommended as a first-line treatment for several cancers in the Chinese Society of Clinical Oncology (CSCO) guidelines, including non-small cell lung cancer (NSCLC), esophageal cancer, hepatocellular carcinoma and Hodgkin’s lymphoma.

Among patients with NSCLC, the progression-free survival (PFS) was significantly prolonged in those receiving camrelizumab in combination with chemotherapy, compared to those who received chemotherapy alone [10]. Similar results were also found in hepatocellular carcinoma and esophageal squamous cell carcinoma [11–13]. The majority of patients who are received camrelizumab beneficial effects from the treatment. However, some patients develop immune resistance during monotherapy with camrelizumab. Currently, it is evident in other therapeutic areas that ADA can significantly influence treatment outcomes [14–16], yet there are relatively few studies examining the impact of ADA on immunotherapy in cancer patients. Consequently, the rate of ADA production and its effect on treatment outcomes remain under-explored in patients receiving camrelizumab.

In rheumatology, the production of ADA can be mitigated through immunosuppressive therapies [17, 18]. While this approach is commonly employed in hematological cancers, its applicability in solid tumors is less clear. Some research has shown that patients can still generate ADA even when immunosuppressants are used alongside immunostimulants [19, 20]. Another strategy involves targeting B cells with anti-CD20 drugs. However, in relevant studies, while B cell depletion may occur, it is not yet clear whether this will lead to a decrease in the frequency, titer, or incidence of ADA [21, 22].

The etiology and mechanisms of ADA are multifaceted, involving both patient-related factors and drug-related variables [23]. When mAbs are administered to cancer patients, the development of ADA often correlates with the stage of cancer, with higher ADA levels typically observed in the early stages of the disease. Initially, the molecular basis for ADA was believed to stem from the murine origin of mAbs, which the human immune system recognizes as “non-self.” However, even the use of fully humanized antibody genes does not entirely eliminate immunogenicity and the potential for ADA induction [24]. The dose and duration of mAb therapy also influence the occurrence of ADA, and the incidence varies with ICIs, necessitating a thorough characterization of their clinical significance [4, 25]. For atrizumab, pembrolizumab, dostatuzumab, and avilimumab, the reported maximum rates of ADA positivity were 54.1%, 2.1%, 2.9%, and 5.9%, respectively [26–28].Previous research has demonstrated that patients positive for ADA antibodies progressed sooner than those negative for ADA antibodies after treatment with ipilimumab, nivolumab, or pembrolizumab [29].However, it remains unclear whether the production of ADA will affect the efficacy of camrelizumab treatment in patients with advanced solid tumors.

Since the advent of the first generation of mAbs, immunogenicity has been described decades ago. However, at present, the relationship between ICIs and ADA is not known. Therefore, in this study, we tried to explore the relationship between ADA levels and the therapeutic efficacy of camrelizumab, and to explore the relationship between patients with high ADA levels and patients with low ADA levels, PFS and overall survival (OS). We perform ADA levels testing to help clinicians adjust the dosage or treatment option in a timely manner in patients receiving camrelizumab.

## Materials and methods

### Patient population and data extraction

This retrospective study gathered blood pressure data from tumor patients who received camrelizumab treatment at the Provincial Hospital Affiliated with Shandong First Medical University between November 2020 and March 2022. This study was conducted in accordance with the Declaration of Helsinki, and the scheme was reviewed and approved by the Medical Ethics Committee of Shandong First Medical University (Approval number: SWYX: NO.2020-304). Eligible patients will provide voluntary informed consent before the commencement of the study.

The study enrolled patients who were 18 years or older, diagnosed with cancer through histological or cytological analysis. All patients had undergone at least three cycles of induction therapy with camrelizumab monotherapy or in combination (at a dose of 3 mg/kg), with treatments separated by approximately 3-week intervals. Patients with significant organ dysfunction or failure, who are unable to complete the follow-up procedures, will be excluded from this study. Ultimately, blood samples from 47 patients were included in the analysis for this study.

Two researchers conducted the patients’ data extraction process independently, adhering to the predefined inclusion and exclusion criteria. The following information was extracted for each patient: patient age, sex, cancer type, disease stage at baseline, response to therapy. The primary outcomes included OS and PFS. Our study adhered strictly to the inclusion and exclusion criteria defined by the study design. We collected hematology samples from November 2020 to March 2021 and assigned them to the experimental cohort. After identifying the relationship between ADA levels and camrelizumab treatment outcome, we collected new hematology samples from patients and used the samples collected between January and March 2022 as the validation cohort.

## Blood sampling

Baseline blood samples were collected before the first camrelizumab treatment (baseline), and blood samples for each cycle were collected after the patient received camrelizumab treatment. The patients’ blood samples were collected by venipuncture using a Vacutainer tube (BD Biosciences). The samples were allowed to clot and serum was centrifuged at 1000 × g for 5 minutes and stored at −80°C.

To maintain consistency in timing and mitigate the influence of drug clearance, blood samples collected less than 18 days or more than 24 days after the last infusion were excluded from the analysis of serum drug levels.

## Measurement of ADA and serum levels of camrelizumab

To detect the concentration of ADA, we use camrelizumab (HY-P9971) for coating. The serum was diluted at a ratio of 1:1000. The diluted blood samples were then added to a 96-well plate that had been pre-coated with camrelizumab at a volume of 100 μL per well and were incubated at room temperature for 60 minutes. Following this, the plate was washed 4 times. The 100 μl of horseradish peroxidase (HRP) (HY-P83662) detection reagent was added to each well and the plate was incubated for an additional 30 minutes at room temperature. After a further 5 washes, 100 μl of 3,3′,5,5′-tetramethylbenzidine (TMB) was added to each well. The plate was then incubated at room temperature until the plate changed color. To terminate the reaction, 100 μl of stop solution was added to each well. Absorbance measurements were taken using a BioTek microplate reader at a wavelength of 450 nm.

The concentration of serum camrelizumab was measured using a commercial ELISA according to the manufacturer’s instructions (kit # 200-585-CAM). Antibodies to camrelizumab are pre-coated onto microwells. Serum samples were diluted at a ratio of 1:1000 using WDS and subsequently added to a 96-well. The samples were incubated at room temperature for 1 hour. Following four washes with washing solution, the samples were incubated with HRP conjugate at room temperature for 30 minutes. After an additional five washes, TMB was added to the wells. The reaction was quantified using a BioTek microplate reader.

## Statistical analysis

The independent sample *t*-test was employed to compare the difference in ADA concentration between baseline values and those obtained from blood samples following the third cycle of camrelizumab treatment and beyond. The paired sample *t*-test was utilized to assess the efficacy of camrelizumab in patients who had received treatment for three cycles or more, and the difference between ADA levels and camrelizumab concentrations. Linear regression was used to compare camrelizumab concentration according to ADA levels. OS is calculated as the duration from the initiation of treatment to the time of death from any cause. PFS is measured as the interval between the commencement of treatment and the occurrence of disease progression or death due to any cause. The survival outcomes were analyzed using the Kaplan-Meier method, and the differences between subgroups were evaluated with the log-rank test. Multivariate Cox proportional hazards models were employed for the analysis of OS and PFS. The primary outcomes of OS and PFS were assessed through the Hazard Ratio (HR), 95% confidence interval (CI), and *P* value. Statistical significance was set at two-sided *P* values<.05. Statistical analysis was conducted using GraphPad Prism 8.0 and IBM SPSS Statistics 27.0 software.

## Results

### Process of inclusion of the included patients

**Figure 1.**
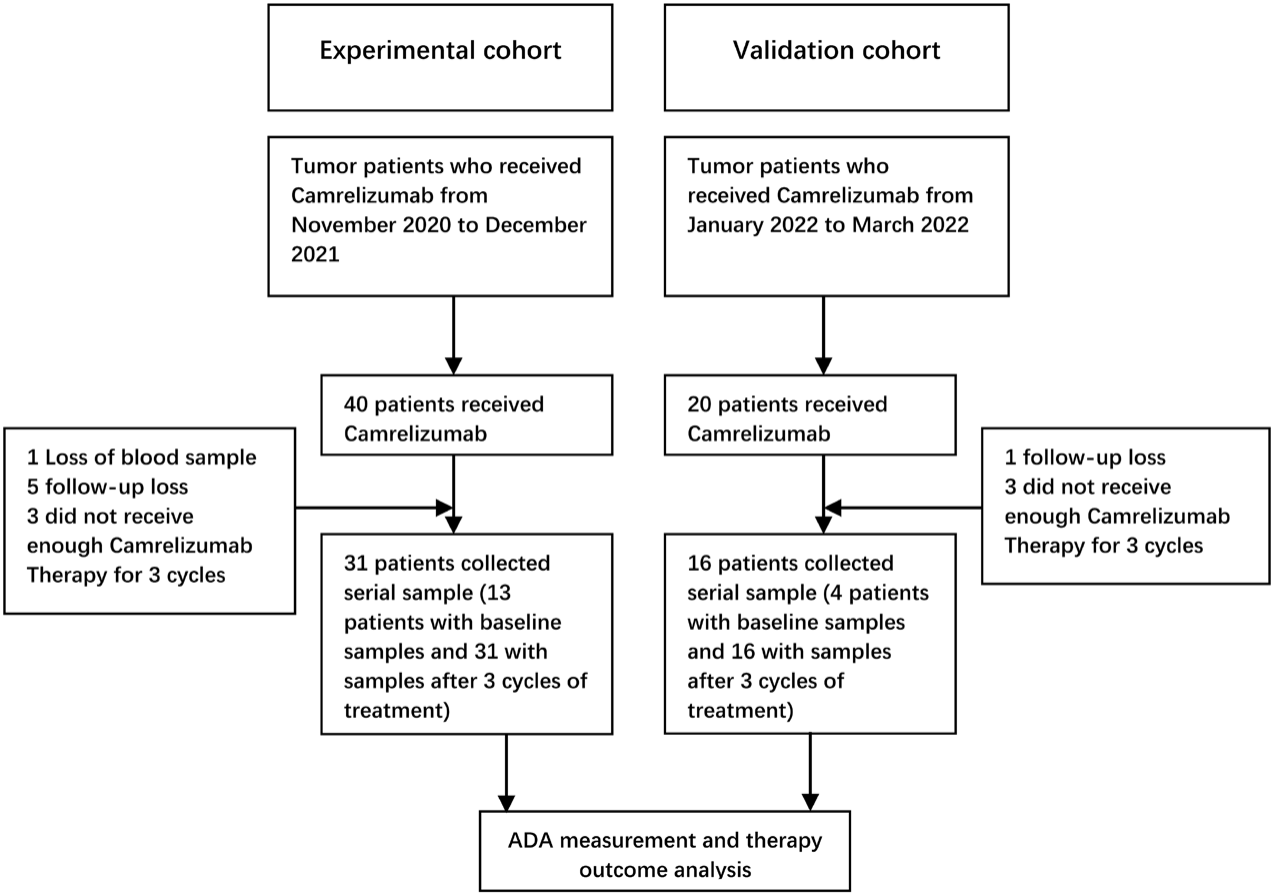
Flowchart of the patient included process. ADA, anti-drug antibody.

From November 2020 to March 2021, we conducted a retrospective analysis of 40 cancer patients who were treated with camrelizumab at the Shandong Provincial Hospital Affiliated to Shandong First Medical University. In the experimental cohort, a total of 31 patients were ultimately enrolled. We initially analyzed the association between ADA levels and treatment efficacy as well as survival outcomes. Between January and March 2022, we included an additional 20 patients, of whom 16 ultimately met our enrollment criteria to constitute our validation cohort. After excluding one blood sample loss, six patients with failed follow-up, and six patients who received less than three treatment cycles, we analyzed the ADA responses of 47 patients (31 from the experimental cohort and 16 from the validation cohort) (Fig. 1).

## The characteristics of the patients

A total of 47 patients were included in this study. The overall median age of the patients included in the study was 62 years old, and the majority of these patients had lung cancer and esophageal cancer. Among these patients, only one had a clinical stage 2 diagnosis, while the remainder had clinical stage 3 or 4 cancer (Table 1). The table below presents the baseline characteristics of the patients in both the experimental and validation cohorts. The median follow-up times for the experimental cohort and the validation cohort were 21.75 months and 28.5 months, respectively.

**Table 1.** The baseline characteristics of the patients in the experimental and validation cohorts

	Experimental cohort	Validation cohort	All
No. of patients (%)	31(66.0)	16(34.0)	47
Age,			
Median (range)	62(34–84)	63.5(30–82)	62(30–84)
Gender (%)			
Male	20 (64.5)	13 (81.3)	33 (70.2)
Female	11 (35.5)	3 (18.7)	14 (29.8)
Cancer types (%)			
Lung cancer	16 (51.6)	6 (37.5)	22 (46.8)
Esophageal cancer	8 (25.8)	7 (43.8)	15 (31.9)
Stomach cancer	2 (6.5)	2 (12.5)	4 (8.6)
Carcinoma of gallbladder	1 (3.2)	–	1 (2.1)
Hepatocellular carcinoma	2 (6.5)	–	2 (4.3)
Breast cancer	1 (3.2)	–	1 (2.1)
Colorectal cancer	1 (3.2)	–	1 (2.1)
Cholangiocarcinoma	–	1 (6.2)	1 (2.1)
Disease stage at baseline (%)			
II	1 (3.2)	–	1 (2.1)
III	15 (48.4)	5 (31.2)	20 (42.6)
IV	15 (48.4)	11 (68.8)	26 (55.3)
Response to therapy (%)			
PD	10 (32.3)	4 (25.0)	14 (29.8)
SD	9 (29.0)	8 (5.0)	17 (36.2)
PR	12 (38.7)	4 (25.0)	16 (34.0)
ADA level (%)			
High level (≥1200 ng/ml)	21(67.7)	7(43.6)	28 (59.6)
Low level (<1200 ng/ml)	10(32.3)	9(56.4)	19(40.4)

ADA, anti-drug antibody; PD, progressive disease; PR, partial response; SD, stable disease.

## The level of ADA concentration at baseline and three cycles

**Figure 2.**
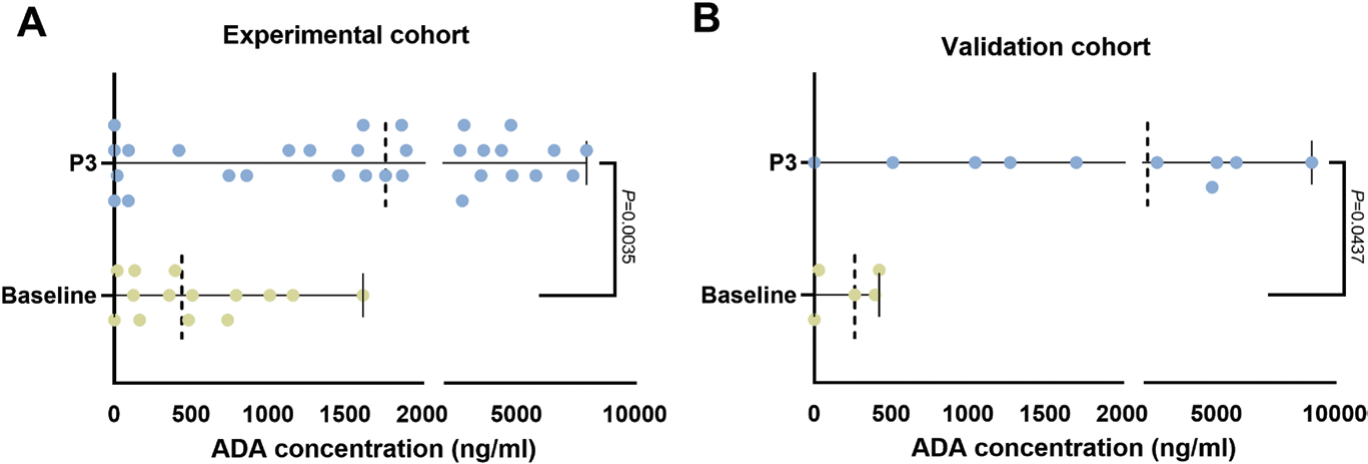
The level of ADA concentration in patients who had been treated (three cycles of camrelizumab treatment: P3) or not treated (baseline) with camrelizumab. (A) ADA levels were compared between baseline and after three cycles of camrelizumab treatment in the experimental cohort. (B) ADA levels were compared between baseline and three cycles of camrelizumab treatment in the validation cohort. ADA, anti-drug antibody; P3, 3 cycles.

Blood samples were collected after patients had not been treated with camrelizumab (Baseline) and received three cycles of camrelizumab treatment (P3). We found that, in comparison to the initial ADA levels, the ADA levels were significantly elevated in patients who after three cycles of camrelizumab therapy in experimental cohort (baseline mean vs P3 mean: 535.4 vs 2428; 95% CI: 656.3–3129; *P* = .0035) (Fig. 2A). We also obtained similar results in the validation cohort (baseline mean vs P3 mean: 221.8 vs 3172; 95% CI: 97.63–5804; *P* = .0437) (Fig. 2B).

## The ADA levels of the dynamic blood samples of the patients

**Figure 3.**
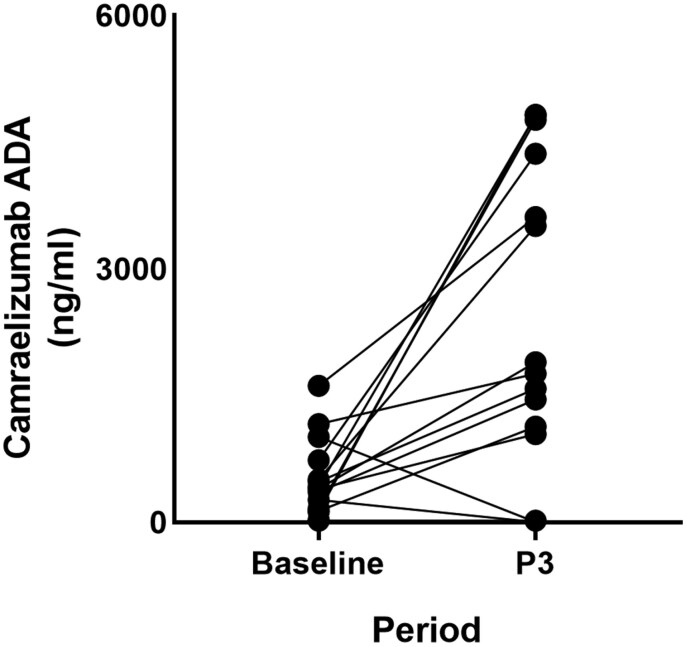
The dynamic blood samples of the patients were measured for ADA levels, specifically the baseline ADA levels prior to treatment with camrelizumab and the ADA levels after three treatment cycles with camrelizumab. ADA, anti-drug antibody; P3, 3 cycles.

Blood samples were obtained from 17 patients both before and after they had undergone three cycles of treatment with camrelizumab. Upon testing the collected samples for ADA levels responses, it was observed that the majority of patients (16 patients, ADA level<1200 ng/ml) exhibited no or low detectable ADA in their serum at baseline. Only two patients had higher ADA levels at baseline compared to three cycles. Conversely, after three cycles of camrelizumab treatment, the ADA levels in the majority of patients (15 patients) were significantly higher than their baseline values (*P* = .0014) (Fig 3).

## The level of ADA concentration and evaluation of therapeutic efficacy

**Figure 4.**
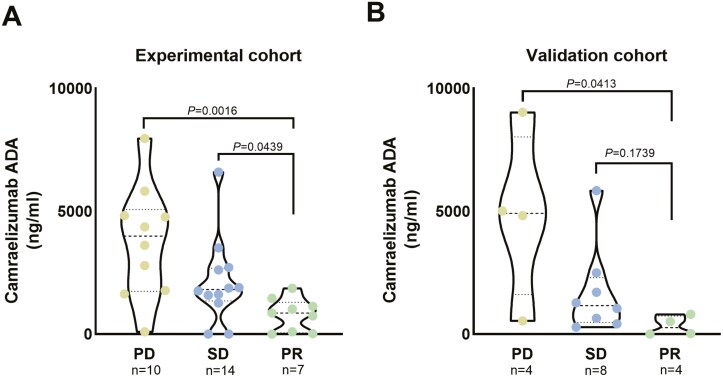
The therapeutic efficacy of patients treated with camrelizumab was compared in the experimental cohort and the validation cohort, respectively. (A) The relationship between therapeutic efficacy and ADA levels after three cycles of Camrelizumab treatment in the experimental cohort. (B) The relationship between therapeutic efficacy and ADA levels after three cycles of Camrelizumab treatment in the validation cohort. ADA, anti-drug antibody; PD, progressive disease; PR, partial response; SD, stable disease.

We further assessed the efficacy of patients who had completed three cycles of camrelizumab treatment. Patients were classified into three groups based on the evaluation of therapeutic efficacy: progressive disease (PD), partial response (PR), and stable disease (SD). Our findings revealed that the ADA concentration after three cycles of Camrelizumab treatment in the PR group differed from that in the PD group (*P* = .0016). Similarly, it significantly differs from the SD group (*P* = .0439) (Fig. 4A). This pattern was also observed in the validation cohort, where the ADA concentration after three cycles of Camrelizumab treatment in the PD group was also found to differ from that in the PR group (*P* = .0413). However, only a trend, but without statistically significant difference was found between PR and SD (*P* = 0.1739), potentially due to limited sample size. (Fig. 4B).

## Correlation of serum levels of ADA and camrelizumab after three cycles of camrelizumab treatment

**Figure 5.**
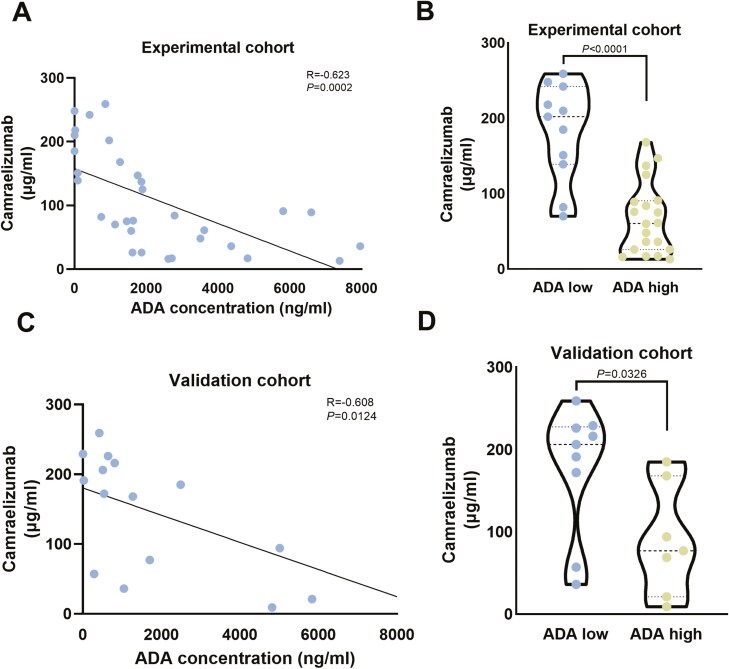
Correlation between serum camrelizumab concentration and ADA levels after three cycles of camrelizumab treatment in patients. (A) Correlation between serum camrelizumab and ADA concentration in the experimental cohort. (B) Correlation between serum camrelizumab and ADA concentration in the validation cohort. (C) Serum camrelizumab concentrations were compared based on ADA levels in the experimental cohort. (D) Serum camrelizumab concentrations were compared based on ADA levels in the validation cohort. ADA, anti-drug antibody.

To further elucidate how high ADA levels after three cycles of camrelizumab treatment affect the immunotherapy efficacy of camrelizumab, we evaluated serum camrelizumab concentrations based on ADA status. After three cycles of treatment, camrelizumab concentration was negatively correlated with ADA levels (experimental cohort: *R*^2^ = 0.3876; validation cohort: *R*^2^ = 0.3702) (Fig. 5 A and C). In patients with a high level of ADA, serum camrelizumab concentrations were significantly reduced after receiving three cycles of camrelizumab. In order to minimize the possibility of false positives, ADA levels of 1200 ng/ml or higher were defined as high ADA levels. We found similar results in both the experimental and validation cohorts, with significantly reduced camrelizumab concentrations in serum in patients receiving three cycles of camrelizumab in patients with high ADA levels (Experimental cohort: *P*<.0001; Validation cohort: *P* = .0326) (Fig. 5B and D).

## The relationship between the ADA levels and survival analysis

**Figure 6.**
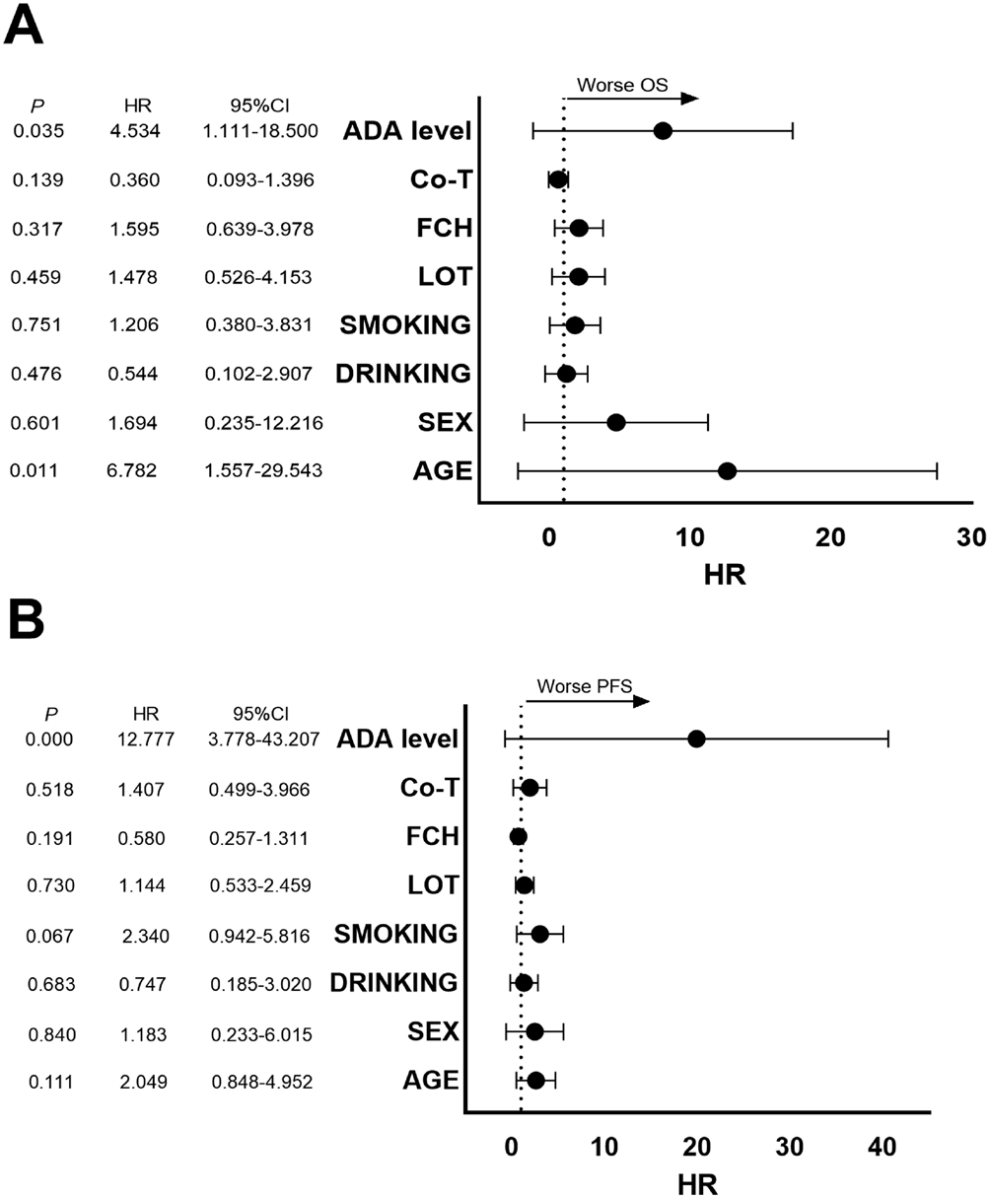
Multivariate Cox regression analyses of multiple variables for OS and PFS in cancer patients treated with camrelizumab. (A) Multivariate Cox regression analysis of cancer patients OS treated with camrelizumab. (B) Multivariate Cox regression analysis of cancer patients PFS treated with camrelizumab. ADA, anti-drug antibody; CI, confidence interval; Co-T, co-treatment; FCH, Family Cancer History; HR, Hazard Ratio; LOT, Lines of Therapy; OS, overall survival; PFS, progression-free survival.

For survival analysis, patients were divided into two groups based on ADA concentration after three cycles of camrelizumab treatment: the “ADA High” group and the “ADA Low” group. Out of the 47 patients included in the study, 28(59.6%) were classified as ADA High. Given the impact of ADA on immunotherapy efficacy and safety, we proceeded to integrate them with other measures for a comprehensive prediction. Initially, we assessed the multivariate differences of the included indicators. In a multivariate analysis of our study cohort, we found that high ADA levels (≥1200 ng/ml) were associated with shorter PFS and OS (Fig 6).

**Figure 7.**
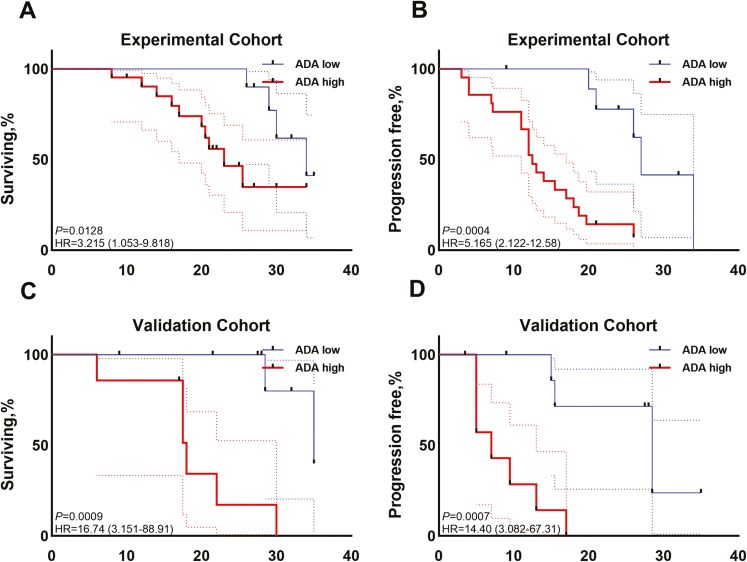
OS and PFS by ADA levels after three cycles of camrelizumab treatment. (A and B) The (A) OS and (B) PFS of ADA High level and ADA Low level in experimental cohort are illustrated. (C and D) The OS (C) and (D) PFS of ADA High level and ADA Low level in validation cohort are illustrated. ADA, anti-drug antibody; HR, Hazard Ratio.

In the experimental cohort, patients with ADA High level in experimental cohort significantly shorter OS (*P* = .0128), with a median OS of 20.5 months (range: 8–34 months), compared to patients with ADA Low level, who had a median OS of 29.5 months (range: 26–35 months) (Fig. 7A). Additionally, the median PFS for patients with ADA High level was 12.5 months (range: 3–26 months), which was significantly different from the median PFS of 25 months (range: 9-34 months) for patients with ADA Low level in the experimental cohort(*P* = .0004) (Fig. 7B). Similarly, in the validation cohort, we observed similar patterns. Patients in the ADA High level group demonstrated significantly shorter OS (*P* = .0009), with a median OS of 17.5 months (range: 6–30 months), compared to those in the ADA Low level group, who had a median OS of 28.5 months (range: 9–35 months) (Fig. 7C). Furthermore, the median PFS for patients with high ADA levels was 7 months (range: 5–17 months), a significant difference from the median PFS of 27.5 months (range: 3.5–35 months) for patients with low ADA levels (*P* = .0007) (Fig. 7D).

## Discussion

Camrelizumab is the fifth PD-1 inhibitor to be approved in China for immunotherapy. In 2019, it was validated for the treatment of relapsed or refractory classical Hodgkin’s lymphoma in China, having received at least two lines of systemic chemotherapy. In 2020, the State Drug Administration approved its use for the treatment of advanced hepatocellular carcinoma that had previously undergone treatment with Lenvatinib and/or Oxaliplatin-based chemotherapy. Currently, camrelizumab is considered the standard of therapy for various cancers, including lung cancer, hepatocellular carcinoma, esophageal cancer and Hodgkin’s lymphoma [30, 31]. Preclinical studies have demonstrated its high affinity for PD-1, resulting in sufficient clinical efficacy with favorable tolerability [32].

The evidence suggests that few patients who are treated with camrelizumab monotherapy experience long-term beneficial responses and ultimately become resistant to the drug. Upon the introduction of the first generation of mAbs, concerns about immunogenicity were immediately raised. The immunogenicity triggered by ADA could manifest as straightforward pharmacokinetic alterations or escalate to severe infusion reactions due to hypersensitivity [33]. Furthermore, research has indicated that patients who generate ADA often exhibit a more poor clinical response compared to those who do not produce it [34]. Regarding the production of ADA, the presence of a non-human drug, such as animal-derived antiserum, often results in the production of high levels of neutralizing antibodies after a single administration. This response also generates memory cells, which can lead to long-lasting immunity. In contrast, when receiving humanized drugs, the development of ADA requires extended exposure [35]. Therefore, in patients treated with camrelizumab, the mechanisms by which ADA is produced and its impact on them remain unclear.

Previous research has indicated that the production of ADA is associated with the duration of patient treatment [29, 36–38]. Therefore, in this study, we focused on hematological samples from patients who had received at least three cycles of camrelizumab therapy. Of the 47 patients treated with camrelizumab for three or more cycles, 28 (59.6) exhibited a robust ADA response. We subsequently analyzed patient efficacy and concluded that high ADA levels after three cycles may have detrimental effects on patient outcomes (PD vs PR, *P* = .0016; PR vs SD, *P* = 0.439). Similar results were observed in the validation cohort (PD vs PR, *P* = .0413). More significantly, our study found that high ADA levels after three cycles of camrelizumab treatment were statistically associated with significantly reduced OS (*P* = .0128) and PFS (*P* = .0004), with comparable findings in the validation cohort (OS, *P* = .0009; PFS, *P* = .0007). These findings suggest that early detection of high ADA levels after three cycles of camrelizumab treatment may indicate a potential reduction in the immunotherapeutic efficacy of camrelizumab.

In prior studies, Kverneland and his colleagues observed that the presence of ADA in 26% of patients treated with ipilimumab was linked to shorter survival rates in metastatic melanoma patients receiving ipilimumab [29]. Similarly, Chan and his colleagues, analyzing hepatocellular carcinoma patients treated with Atezolizumab, found that 17.4% of patients exhibited elevated ADA levels and concluded that these high levels may reduce the exposure to Atezolizumab and consequently weaken its anticancer effect [38]. In our research, we investigated the outcomes of pan-cancer patients treated with camrelizumab, including those with six prevalent types of cancer: lung, esophageal, gastric, hepatocellular, cholangiocarcinoma, and breast cancer. Our findings echo those of previous studies, indicating that in the early stages of monoclonal antibody treatment, patients tend to produce higher ADA levels. We also found that ADA levels after three cycles of camrelizumab treatment were found to be negatively correlated with serum camrelizumab concentrations. Moreover, high ADA levels after three cycles of camrelizumab treatment potentially compromise the efficacy and survival rates of camrelizumab.

In addition, it is noteworthy that the ADA levels who were treated with camrelizumab for three cycles decreased relative to their baseline levels, ruling out potential influencing factors such as procedural and sample handling variations. However, for ADA levels after three cycles of camrelizumab treatment, two patients showed no significant change in the ADA levels compared to the baseline, suggesting that ADA levels may exhibit a minor degree of fluctuation within patients. Other possible reasons: it is also possible that patients already have ADA present before they undergo treatment with camrelizumab, or the presence of immunoglobulins in the patients before treatment.

Due to limited sample size, this study cannot conclusively validate our results; rather, it should be considered observation research. Additionally, the absence of standardized testing and a unified approach for classifying patient antibodies as positive prevents us from establishing a definitive definition of high ADA levels based on this study alone. Consequently, there is currently a lack of widespread consensus and recognition regarding ADA. This situation hampers the development of guidelines for the appropriate follow-up of patients with positive antibody responses. Moreover, individual patient responses to antibodies varied significantly.

Despite the limitations of this study, we have determined that patients tend to exhibit higher levels of ADA during the early stages of monoclonal antibody therapy. Furthermore, high ADA levels may diminish the efficacy and survival rate of camrelizumab. Future investigations should involve larger, prospective studies to validate these findings. Additionally, early detection of ADA levels may assist clinicians in predicting the potential efficacy of camrelizumab in patients and in making timely decisions regarding dosage adjustments or the transition to alternative treatment options.

## Conclusion

In this study, we observed a correlation between high ADA levels after three cycles and reduced OS and PFS among patients receiving camrelizumab for more than three cycles. We noted that patients exhibited higher ADA levels during the early phase (first three cycles) of camrelizumab treatment. In addition, it found that the higher the ADA concentration, the lower the serum camrelizumab concentration, indicating that there be a negative correlation between the two. These findings warrant further validation through prospective studies in the future.

## Data Availability

Data will be made available on request.
